# Electrodeposition of Polymer Electrolyte Into Porous LiNi_0.5_Mn_1.5_O_4_ for High Performance All-Solid-State Microbatteries

**DOI:** 10.3389/fchem.2018.00675

**Published:** 2019-01-23

**Authors:** Girish D. Salian, Chrystelle Lebouin, Alina Galeyeva, Andrey P. Kurbatov, Thierry Djenizian

**Affiliations:** ^1^Electrochemistry of Materials Research Group, Aix Marseille Univ, CNRS, MADIREL, Marseille, France; ^2^Department of Chemistry and Chemical Technology, Al Farabi Kazakh National University, Alma Ata, Kazakhstan; ^3^Mines Saint-Etienne, Department of Flexible Electronics, Center of Microelectronics in Provence, Gardanne, France

**Keywords:** electropolymerization, lithium nickel manganese oxide, porous materials, polymer electrolyte, Li-ion microbatteries

## Abstract

We report the electrodeposition of polymer electrolyte (PMMA-PEG) in porous lithium nickel manganese oxide (LiNi_0.5_Mn_1.5_O_4_) cathode layer by cyclic voltammetry. The cathode-electrolyte interface of the polymer-coated LNMO electrode has been characterized by scanning electron microscopy and electrochemical techniques. Electrochemical measurements consisting of galvanostatic cycling tests and electrochemical impedance spectroscopy revealed a significant improvement of the capacity values and the increase of the operating voltage. These effects are attributed to the total filling of pores by the electrodeposited polymer that contributes to improve the reversible insertion of Li^+^. A complete all-solid-state microbattery consisting of electropolymerized LNMO as the cathode, a thin polymer layer as the electrolyte, and TiO_2_ nanotubes as the anode has been successfully fabricated and tested.

## Introduction

Due to the highest electrochemical performance of the known energy storage systems, Lithium Ion Batteries (LIBs) have attracted attention to provide energy for low power microelectronic devices like Microelectromechanical Systems (MEMS), smart cards, radio-frequency identification (RFID) tags, biomedical *in vivo* micromachines, etc. However, downscaling the size of batteries alters their properties as the technology relies on the successive deposition of dense thin-films. In order to circumvent this issue, several strategies have been investigated including the use of nanostructured and porous materials (Shaijumon et al., 2010; Arthur et al., 2011; Roberts et al., 2011; Ellis et al., 2014; Ferrari et al., 2015) as well as the modification of the electrode surfaces (Lopez et al., 2014; Ortiz et al., 2016a,b). To improve the energy density of LIB microbatteries, thin-film cathodes with large capacity and/or high working voltage have to be envisioned. Among them, LiNi_0.5_Mn_1.5_O_4_ (LNMO) is interesting due to its high capacity, good cycling stability, and good rate capability, which make it promising as thin-film positive electrode for microbatteries (Kang et al., 2006; Sun et al., 2009, 2012; Fergus, 2010; Ohzuku et al., 2011).

Miniaturizing LIBs also requires the use of solid electrolyte (polymers or glassy materials) because liquid counterparts are responsible for leakage risks and safety issues (Fergus, 2010). Reports of gel polymer electrolyte (GPE) used as electrolytes with LNMO have been reported previously (Bernhard et al., 2013; Sun et al., 2014; Chen et al., 2015). The main objective for the usage of gel polymer electrolyte is to limit the decomposition of the liquid electrolytes and the formation of a Solid Electrolyte Interphase (SEI) at the electrode surfaces hindering the electrochemical reactions. Moreover, manganese present in the cathode materials tends to dissolve in organic liquid electrolyte, especially at elevated temperature (Prabakar et al., 2012; Li et al., 2013). Hence, the high chemical stability of GPEs is interesting to be used as electrolyte materials particularly for cells involving LNMO.

Due to the stability in a wide potential window, polyethylene oxide (PEO) based electrolytes have been identified as potential polymer electrolytes for all-solid-state batteries (Goodenough and Singh, 2015; Long et al., 2016; Cheng et al., 2018). Of late polymethyl methacrylate—polyethylene glycol (PMMA-PEG) has been reported to be an efficient polymer electrolyte for the fabrication of complete all-solid-state microbatteries (Plylahan et al., 2014). Here, PEG serves as the ionic medium for the Li ion conduction while PMMA contributes to the mechanical stability. To improve the performance of the LIBs that employ polymer electrolytes, it is crucial to enhance the surface contact established between the porous electrodes and the electrolyte. The electrodeposition technique is an effective bottom-up approach to optimize the electrode/electrolyte interface. Previous reports have reported the conformal electrodeposition of this polymer electrolyte in self-organized titania nanotubes (TiO_2_ nts) and its benefit on the capacity of the microbattery (Kyeremateng et al., 2011; Plylahan et al., 2012, 2015). We recently showed the positive influence of this approach for a microbattery based on polymer electropolymerized in TiO_2_ nts and LNMO (Salian et al., 2017). While the electrodeposition of the polymer electrolyte in TiO_2_ nts allows the improvement of the cell capacity, applying the same treatment to the LNMO cathode revealed the doubling of the capacity and an increase of the energy density due to a slight increase of the operating voltage. In this work, we study in details the electrodeposition of PMMA-PEG in porous LNMO to get a better insight into the key role of the interface on the performance of all-solid-state Li-ion microbatteries.

## Experimental

### Synthesis of LNMO Cathode and TiO_2_ nts Anode

Porous LNMO serving as cathode was synthesized by a sol-gel technique as described by Cui et al. (2011). The synthesized LNMO was then mixed with carbon black (Super P) and Polyvinylidene fluoride (PVDF) in the ratio of 75:15:10 and grounded in a mortar for 15 min. The ground powder was then mixed with N-methyl-2-pyrrolidone (NMP) to obtain a paste that was subsequently spread on an aluminum disk with a diameter of 8 mm. The electrode was dried under vacuum at 110°C for 8 h.

The TiO_2_ nts serving as anode was synthesized by the anodization of a titanium foil. Before anodization, the Ti foils (99.6% purity Goodfellow and 0.125 mm thickness) were cleaned and sonicated in acetone, isopropanol, and methanol for 10 min each. After drying the foils under compressed air, anodization of the cleaned Ti foil was carried out in an electrolyte containing 96.7 wt. % glycerol, 1.3 wt. % NH_4_F, and 2 wt. % water. A constant voltage of 60 V was applied between the Ti substrate and a Pt foil (counter electrode) for 3 h using a generator (ISO-TECH IPS-603). Then, the sample was washed with deionized water and dried using compressed air. The as-formed TiO_2_ nts were annealed in air at 450°C for 3 h to form the anatase phase. The TiO_2_ nts obtained with these parameters exhibit a thickness of *c.a* 1.5 μm and an outer diameter of 100 nm as it has been reported elsewhere (Plylahan et al., 2015).

### Electropolymerization (EP) of the Electrolyte Into LNMO

The electrodeposition of PMMA-PEG in porous LNMO was performed by cyclic voltammetry (CV) in a three-electrode system using a VersaSTAT 3 potentiostat (Princeton Applied Research), Ag/AgCl (3M KCl) as the reference and Pt electrode as the counter electrode in an aqueous solution containing 0.5 M of bis(trifluoromethanesulfone)imide (LiTFSI) and 0.5 M MMA-PEG (methyl methacrylate-polyethylene glycol) with an average molecular weight of 500 g mol^−1^ [MMA-PEG (500)]. Prior to electropolymerization, the aqueous solution containing the polymer electrolyte was purged with Argon gas for 10 min to remove dissolved oxygen. The cyclic voltammetry experiments were carried out at room temperature for 5, 10, 25, 50, and 100 cycles at the scan rate of 10 mV s^−1^ in the potential window of −0.35 to −1 V vs. Ag/AgCl (3 M KCl). The electropolymerized LNMO electrode was then dried in vacuum at 70°C for 18 h. In this work, the number of electropolymerization cycle will be referred to as “x EP cycles.” The electropolymerized LNMO at “x EP cycles” is designated as LNMO_(xEP)_, where x = 5, 10, 25, 50, and 100 EP cycles.

### Material and Electrochemical Characterizations

For electrochemical experiments performed in Swagelok-type half cells, the electrolyte consisted in two sheets of gel polymer that were prepared by soaking a Whatman paper disk in 70 μL of a solution of 0.5 M LiTFSI in 2 ml MMA-PEG (500) and then drying them overnight at 70°C under vacuum. For the CV and Electrochemical Impedance Spectroscopy (EIS) measurements on bare and electropolymerized LNMO, the samples were assembled in a three-electrode configuration with two lithium foils serving as reference and counter electrodes. CV measurements were performed in the range of 2.7–4.8 V at different scan rates (0.1, 0.2, 0.75 , and 1 mV s^−1^) successively for 5 cycles each. EIS measurements were performed in the range of 10 mHz−100 kHz with an amplitude of 10 mV at Open Circuit Voltage (OCV). For electrochemical experiments performed in Swagelok-type full cells, electropolymerized LNMO and as-formed TiO_2_ nts electrodes were assembled in a two-electrode configuration using a Whatman paper soaked with the polymer electrolyte. To fabricate the full all-solid-state microbattery, 7 μl of the polymer electrolyte was drop casted at the surface of the TiO_2_ nts electrode. The sample was then dried again at 70°C for 18 h to obtain a homogeneous polymer thin film. The electropolymerized LNMO that was prior deposited on the aluminum disk was then pressed together with the polymer-coated TiO_2_ nts. The wire connections were established at the backside of each current collector using a silver conductive paste. All electrochemical cells were assembled in an argon filled glovebox (MBraun, Germany) with <0.5 ppm H_2_O and <0.5 ppm O_2_ atmosphere.

Galvanostatic charge/discharge profiles and EIS measurements were performed using a VMP3 potentiostat-galvanostat (Bio Logic). The Scanning Electron Microscopy (SEM) images were obtained using a CARL ZEISS/Ultra 55 Scanning electron microscope.

## Results and Discussions

**Figure 1:**
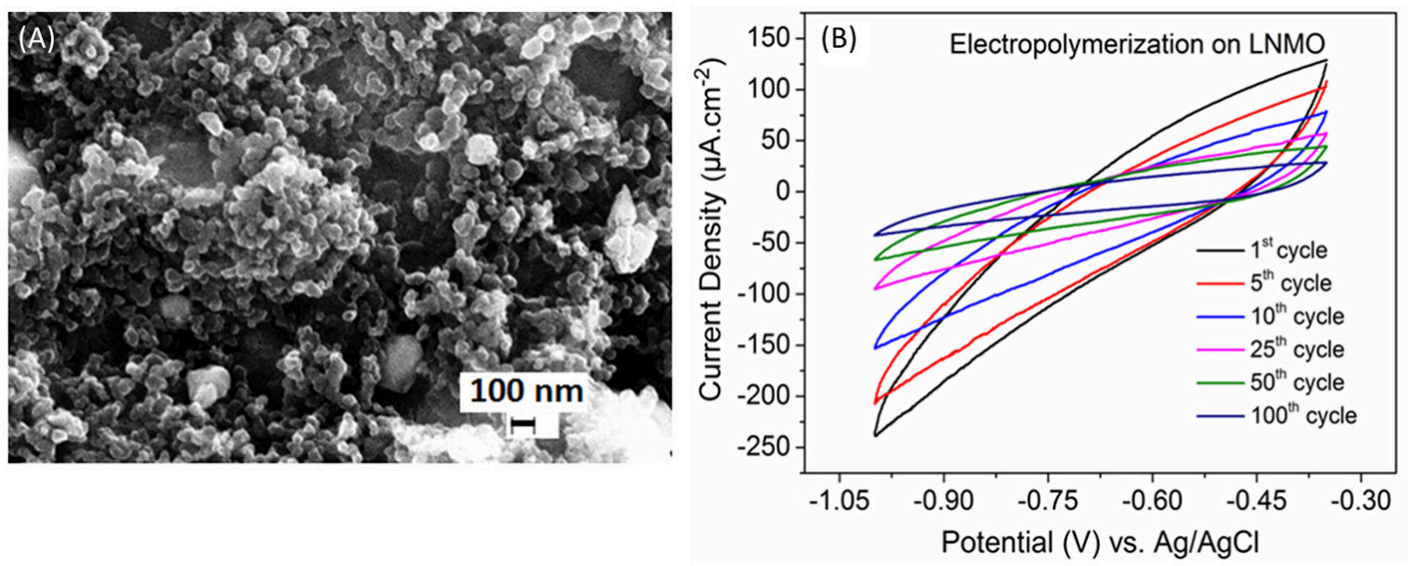
**(A)** SEM image of the synthesized porous LNMO powder. **(B)** Cyclic voltammograms of the PMMA-PEG electropolymerization on LNMO in the potential window −0.35 to −1 V at a scan rate of 10 mV s^−1^.

Figure 1A shows the SEM images of the as synthesized LNMO powder. The particle size varies from 100 nm to 1 μm. The powder is characterized by porous morphology. This is an advantage as pores promote the percolation of the electrolyte (polymer/liquid) in the bulk material. The electropolymerization of MMA-PEG (500) monomer in the presence of the LiTFSI salt was carried out by cyclic voltammetry. The cyclic voltammograms recorded for LNMO composite cathode electropolymerized up to 100 cycles are shown in Figure 1B. The cathodic current density measured at −1 V vs. Ag/AgCl decreases with the number of cycles. The polymer deposition occurs due to the formation of hydrogen free radicals generated by the reduction of protons as it has been reported for electropolymerization on TiO_2_ nanotubes by our research group (Cram et al., 2002; Plylahan et al., 2012; Salian et al., 2017). Thus, the decrease of the cathodic current density with increment in cycle number can be attributed to the formation of successive thin polymer layers acting as an electronic insulator.

**Figure 2:**
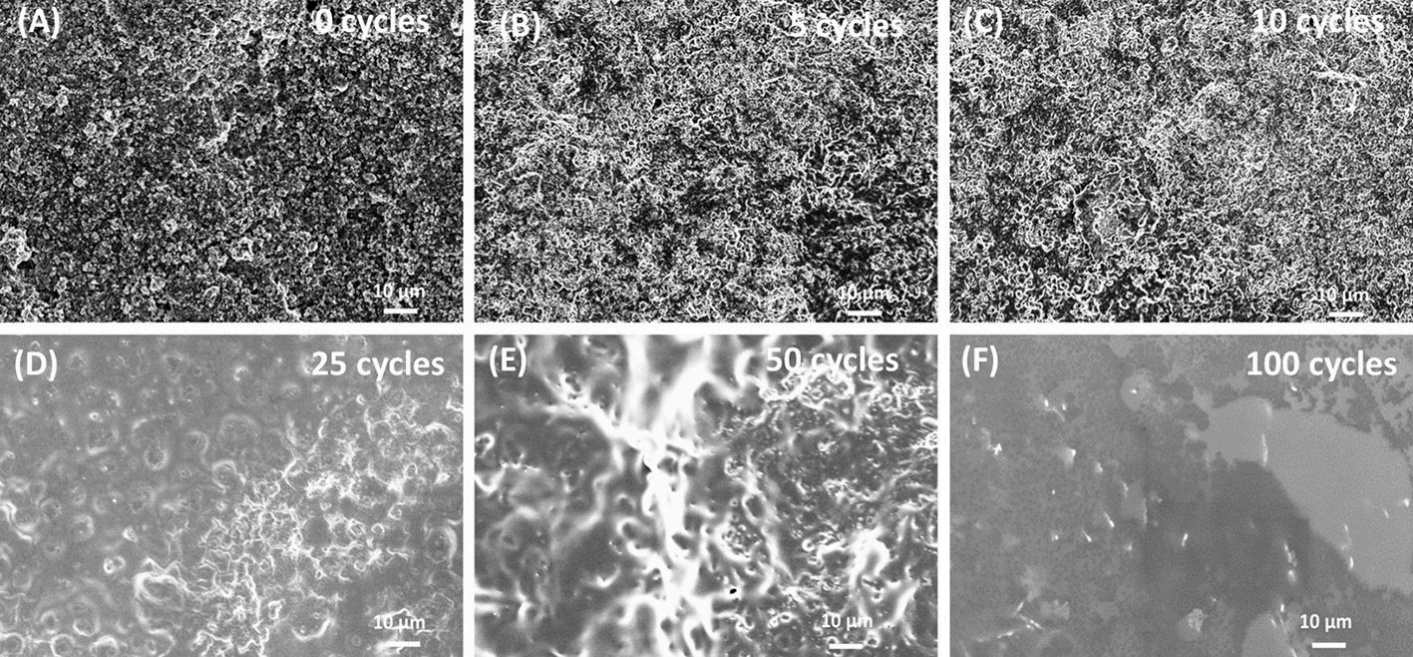
SEM images of electrodeposited PMMA-PEG on porous LNMO by CV at **(A)** 0 cycle; **(B)** 5 cycles; **(C)** 10 cycles; **(D)** 25 cycles; **(E)** 50 cycles; **(F)** 100 cycles.

The relative high cathodic current densities observed for the first cycles are in accordance with the high rugosity of the cathode. This suggests that the polymer layer formed at the initial EP cycles (up to 10 cycles) follows the rugosity of the LNMO electrode (Figure 2A), which is in agreement with the SEM images given in Figures 2B,C. But a thin polymer layer filling the pores has grown beyond 25 EP cycles (Figures 2D,E). For 100 EP cycles, the SEM image reveals that the porous LNMO electrode is completely covered by a smooth layer of polymer (Figure 2F). It can be noted that compared to TiO_2_ nts, the cathodic current density recorded for porous LNMO shows a decrease (*ca*. 80%) with the increasing number of cycles (Salian et al., 2017), which may indicate that the electropolymerization results in a thicker layer than that grown on nanotubes.

**Figure 3:**
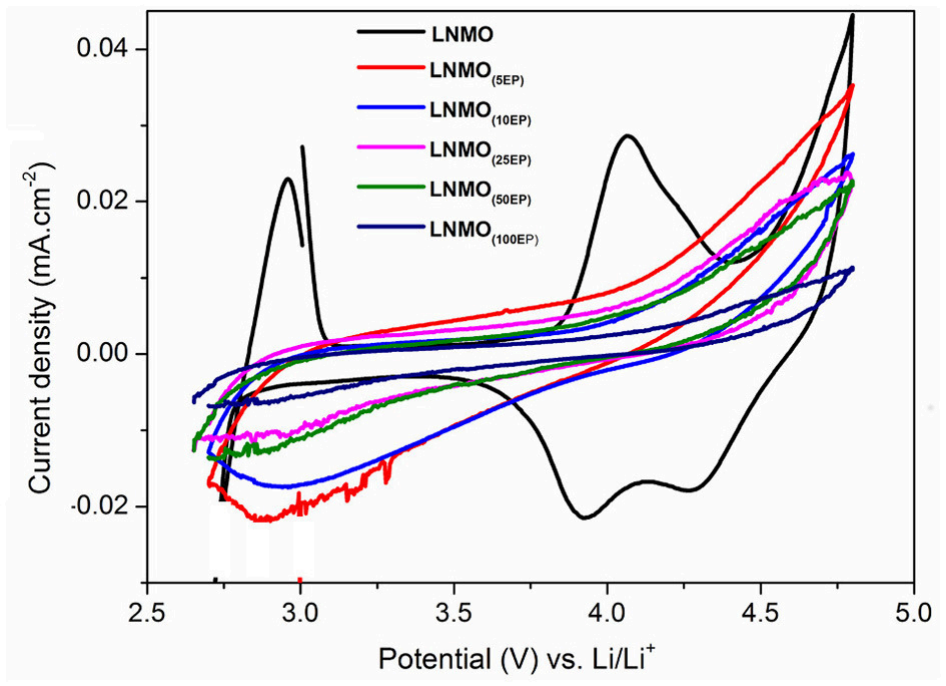
Cyclic voltammograms (1st cycle) of LNMO_(EP)_ samples obtained in the potential range of 2.7–4.8 V at a scan rate of 0.1 mV s^−1^.

The electrochemical reactions of LNMO_(EP)_ samples with Li were studied by cyclic voltammetry. Figure 3 shows the 1st CV curves obtained in a potential range of 2.7–4.8 V vs. Li^+^/Li at a scan rate of 0.1 mV s^−1^. For the bare LNMO sample, the two oxidation peaks appear at 2.9 V and 4.06 V while the reduction peaks are visible at 4.26 V and 3.93 V. These results are similar to the behavior of LNMO in liquid electrolyte for which a main peak attributed to the transition of Mn^3+^/Mn^4+^ is observed at around 4 V and another small peak corresponding to Ni^2+^/Ni^4+^ transition can be found at around 4.7 V (Kunduraci and Amatucci, 2008). The shift in the peaks could be attributed to the lower ionic conductivity of the polymer electrolyte compared to that of liquid electrolyte resulting in ohmic drop effects (Nayak et al., 2014). The CV curves for LNMO_(5EP)_, LNMO_(10EP)_, LNMO_(25EP)_, and LNMO_(50EP)_ show a reduction peak at ~2.95 V, which fades as the number of EP cycles increases. There is an oxidation signal that also fades with the increasing number of EP cycles but no peak is present. Moreover, the LNMO_(100EP)_ shows no significant redox peaks in the CV. The decrease in current densities with increase of the number of EP cycles is consistent with the ohmic drop effect, which is due to the increase of the polymer thickness.

**Figure 4:**
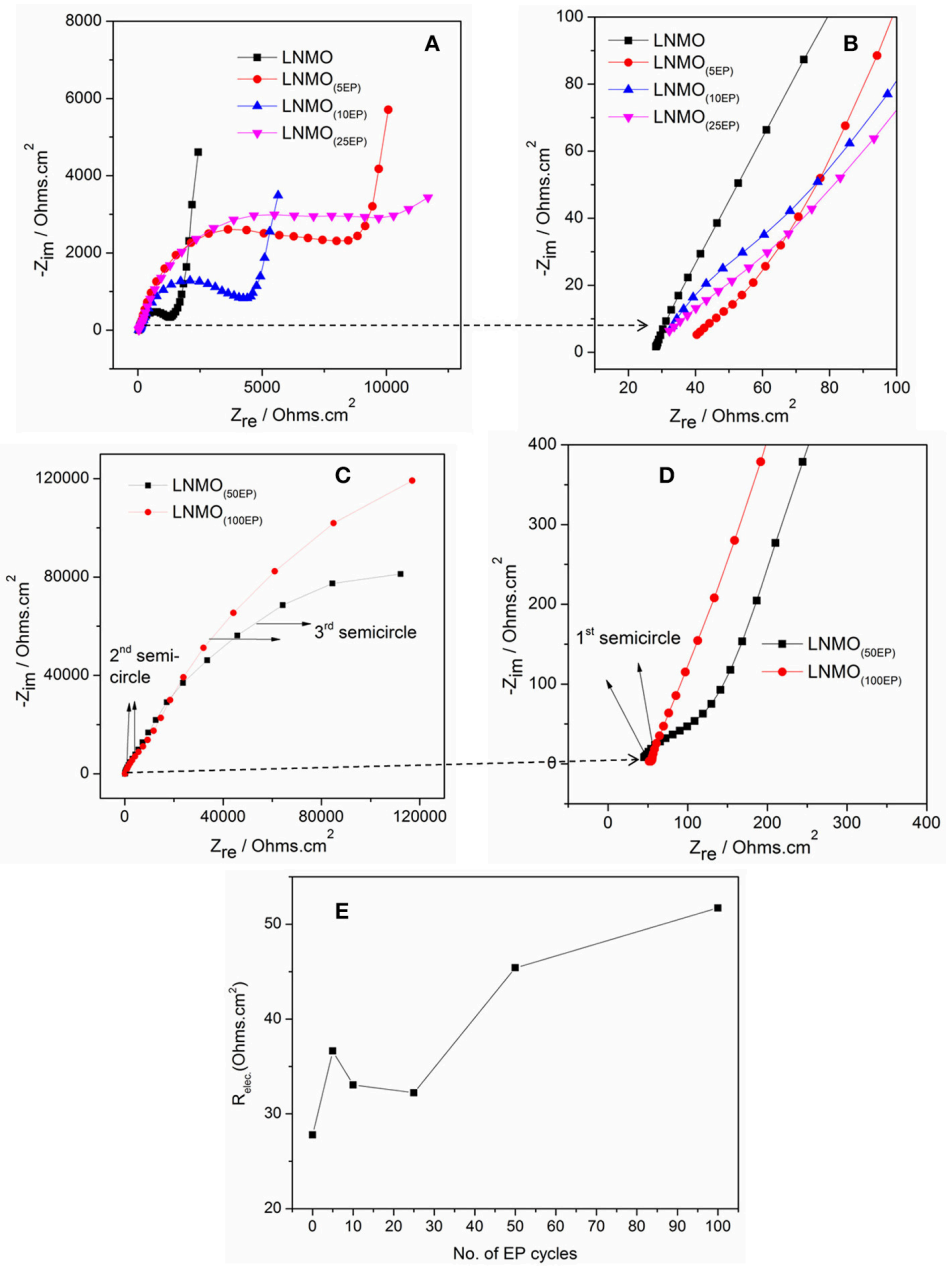
EIS spectra of **(A)** bare LNMO, LNMO_(5EP)_, LNMO_(10EP)_, LNMO_(25EP)_; **(B)** 1st semicircles of the same samples at very high frequencies; **(C)** EIS spectra of LNMO_(50EP)_ and LNMO_(100EP)_; **(D)** 1st semicircles of the same samples at very high frequencies; **(E)** relationship between R_elec_ and the number of EP cycles. The EIS tests were performed at OCV in the frequency range of 100 kHz−10 mHz at an amplitude of 10 mV.

Bare LNMO and LNMO_(EP)_ electrodes were characterized by EIS tests at OCV. Figure 4 shows the EIS plots obtained in a frequency range of 100 kHz−10 mHz at an amplitude of 10 mV. The experimental data were fitted using the equivalent circuit mentioned in Table 1. Here, R_elec_ is the electrolyte resistance that can be expressed by Equation (1):

(1) $$\begin{array}{r} R_{elec}=R_{EP}+R_{separator} \end{array}$$

where R_EP_ and R_separator_ are the respective contributions of the electrodeposited polymer thin layer and the separator (i.e., the polymer embedded in the Whatman paper). R_CEI_ takes into account the formation of a SEI thin film on the LNMO surface, so-called cathode electrolyte interphase (CEI) (Nayak et al., 2014; Kohs et al., 2017). R_CT_ refers to the interfacial property of the charge transfer resistance. Q_CEI_, Q_DL_, and Q are the associated constant phase elements (CPE) with the respective resistances, used in order to account for the porous nature of the electrodes. The equivalent circuit for the EIS spectra of bare LNMO, LNMO_(5EP)_, LNMO_(10EP)_, and LNMO_(25EP)_ (Figure 4A) is [R_elec_ + Q_CT_/(R_CT_+Q)]. The high frequency limit of the impedance intercepts the x-axis and corresponds to the electrolyte resistance. Still at high frequencies, when it decreases, a short slope change is observed (Figure 4B). This might be due to the porous nature of the material or to contact resistances (current collector, interparticles) in the electrode (Lasia, 2002; Levi et al., 2014). The main semicircle in the medium frequency and the sloped line in the lower frequency are attributed, respectively to the charge transfer resistance and the capacitive limit behavior. The equivalent circuit for the EIS spectra of LNMO_(50EP)_ and LNMO_(100EP)_ is [R_elec_ + Q_CEI_/R_CEI_+ Q_DL_/R_CT_]. In addition to the previously mentioned high frequency features, there are 2 semicircles in the medium and low frequencies. The 1st semicircle can be attributed to the resistance due to the CEI formation while the 2nd semicircle can be attributed to the charge transfer resistance at the interface.

**Table 1:** EIS parameters obtained after fitting with the equivalent circuits for the polymer-coated LNMO_(EP)_.

	**R_**elec.**_(Ω.cm^**−2**^)**	**R_**CEI**_ (Ω.cm^**−2**^)**	**R_**CT**_ (Ω.cm^**−2**^)**	**Deviations**	**χ^**2**^ value**
Equivalent circuit	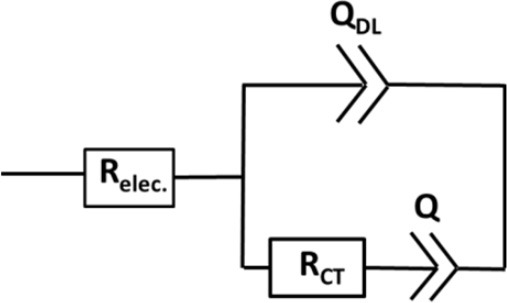				
Bare LNMO	27.79	–	1,387	0.5	0.03
LNMO_(5EP)_	36.64	–	3,811	0.8	0.06
LNMO_(10EP)_	33.06	–	3,097	1.1	0.03
LNMO_(25EP)_	32.24	–	7,620	1.2	0.04
Equivalent circuit	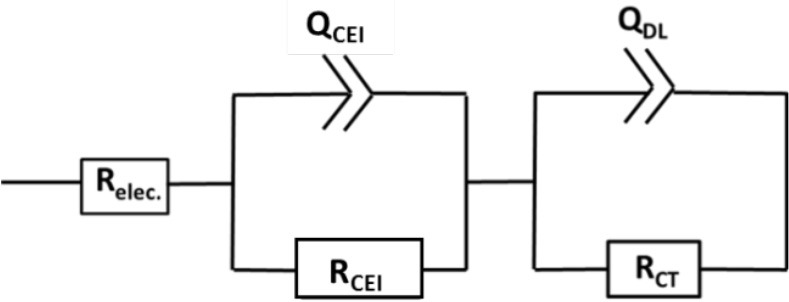				
LNMO_(50EP)_	45.41	1,718	240,000	0.5	0.02
LNMO_(100EP)_	51.72	4,826	421,000	0.7	0.02

Figure 4E shows the evolution of R_elec_ with the number of EP cycles. It globally increases from bare LNMO to LNMO_(100EP)_. However, we note that there is a decrease of R_elec_ between the LNMO_(5EP)_ and LNMO_(25EP)_. For these latter layers, the porosity of the LNMO is not fully filled by the electropolymer. If we consider that R_separator_ is constant, R_elec_ evolution should come from R_EP_, the contribution of the electropolymer deposited after successive EP cycles. For LNMO_(10EP)_ and LNMO_(25EP)_, the electropolymer layer is still very thin so R_EP_ is negligible. But, as it tends to covers the active surface of LNMO, this probably decreases the resistance due to the interface between the electropolymer and the separator. Thereafter, as the electropolymer thin-film grows after the complete pore filling, its thickness becomes less negligible. Thus, R_EP_ and then R_elec_ steadily increases for the LNMO_(50EP)_ and LNMO_(100EP)_.

The semicircle corresponding to R_CEI_ is not seen for bare LNMO, LNMO_(5EP)_, LNMO_(10EP)_, and LNMO_(25EP)_. Or there could be a possible superposition of R_CEI_ with R_CT_ (Aurbach et al., 2002). Thus, very thin CEI layers could be grown on the rough LNMO surface without any specific features on the EIS spectra. In the case of LNMO_(50EP)_ and LNMO_(100EP)_, the first semicircle that appeared at high frequencies was associated to a more prominent contribution of the CEI. Indeed, the formation of CEI could occur as the active surface area of the LNMO is more coated by the polymer. Regarding the charge transfer resistance R_CT_, it increases with the number of EP cycles. It suggests that the kinetic reactions at the interface are limited by the electrodeposited polymer, which is coherent with the evolution of the CV peaks. The large increase in the charge transfer resistance R_CT_ for LNMO_(50EP)_ and LNMO_(100EP)_ could therefore be attributed to the gradual decomposition of the polymer with cycling as it has been suggested (Plylahan et al., 2015).

**Figure 5:**
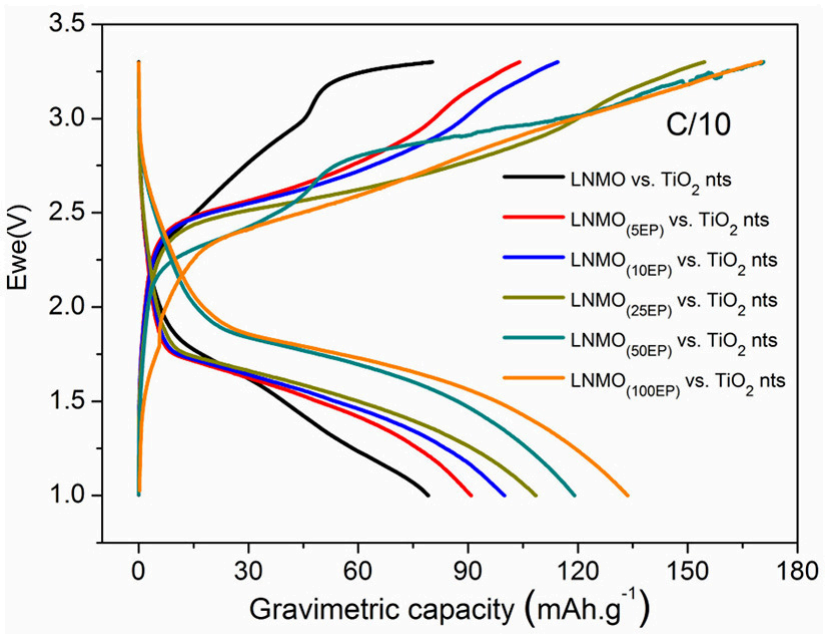
First reversible charge/discharge capacities for LNMO_(EP)_ vs. TiO_2_ nts at C/10 in the potential range of 1–3.3 V.

To investigate the performance of LNMO_(EP)_ electrodes in a full cell configuration, the different LNMO_(EP)_ electrodes were assembled with self-supported TiO_2_ nts negative electrode. Herein the mass limitation is controlled by the TiO_2_ nts. Figure 5 shows evolutions of the first reversible cycle of the full microbatteries.

We can clearly see that the polymer electrolyte deposition has a gradual positive influence on the discharge capacity values of the complete cells. Indeed, the capacity increases from 80 mAh g^−1^ for non-modified electrodes to 134 mAh g^−1^ for LNMO_(100EP)_. Even if the internal resistance of the battery increases with the polymer thickness, the active surface of the material in contact with the electrolyte is significantly enhanced, promoting the reversible insertion of Li^+^. Also remarkably, the operating voltage of the cell is slightly increasing. This effect contributes to the improvement of the energy and power densities.

**Figure 6:**
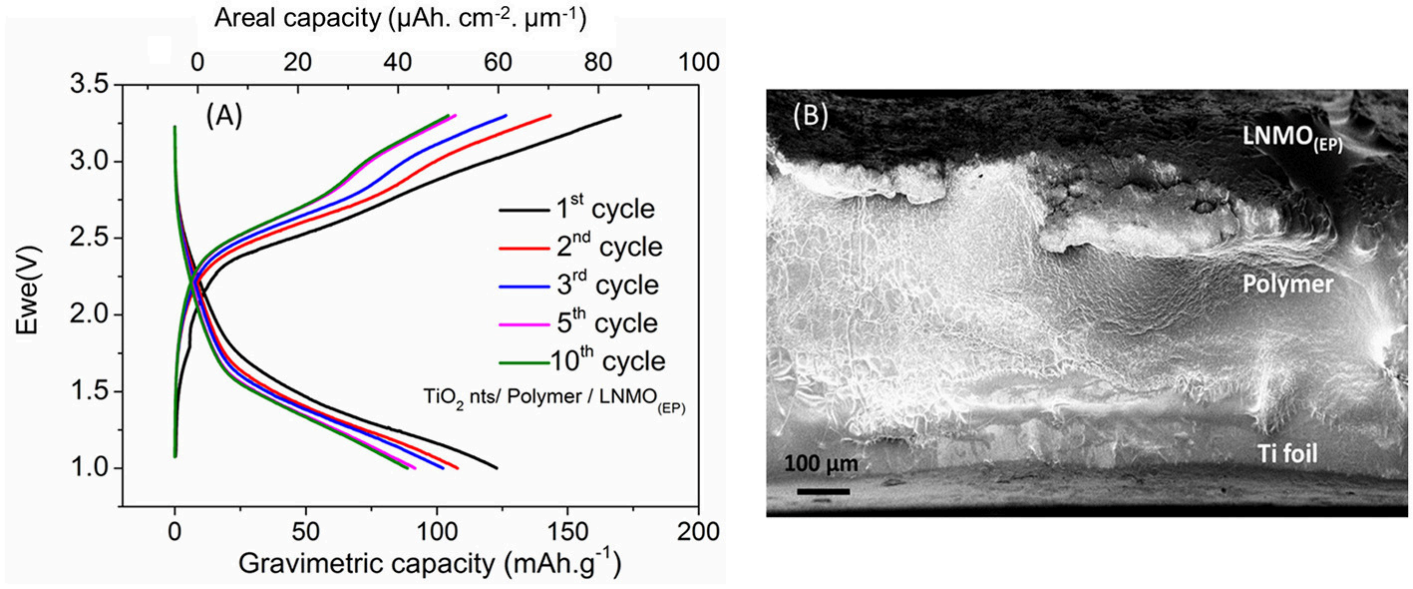
**(A)** Galvanostatic charge/discharge profile of TiO_2_ nts/Polymer/LNMO_(100EP)_ microbattery at C/10 rate; **(B)** cross-sectional SEM image of the all-solid-state battery composed of LNMO_(100EP)/_Polymer/TiO_2_ nts.

The LNMO_(100EP)_ cathode was used to fabricate an all-solid-state microbattery. The microbattery is designated as TiO_2_nts/Polymer/LNMO_(100EP)_. Figure 6A shows the galvanostatic charge/discharge profile of the all-solid-state microbattery in the potential window of 1–3.3 V at C/10 for 10 cycles. Such a microbattery shows an operating voltage at 1.8 V. As the limiting reactant material of the microbattery is TiO_2_ nts, the capacity is reported vs. the anode mass and its geometrical surface. The mass was calculated considering a density of 4.23 g cm^−3^ and a porosity of 60% (Plylahan et al., 2014). The microbattery shows a first discharge capacity of 122 mA h g^−1^ (60 μAh cm^−2^ μm^−1^). The coulombic efficiency (CE) for the first cycle corresponds to 72%. The discharge capacity for the 10th cycles is 89 mA h g^−1^ (44 μAh cm^−2^ μm^−1^). The corresponding CEs from 2nd to 10th cycle are about ~85%. The capacity retention of the microbattery up to the 10th cycle corresponds to 72%. The charge/discharge curves for this self-standing microbattery shows similar characteristic to that of the full cell swagelok tests as discussed in Figure 5. The irreversible capacity loss occurring in the first cycle could be due to the residual water content present in both LNMO and TiO_2_ nts and/or the presence of structural defects trapping irreversibly Li^+^ (Ortiz et al., 2009). The capacity retention for the 10th cycle corresponds to 72%. Figure 6B shows the cross-section image of this microbattery consisting of TiO_2_ nts (1.5 μm long) supported on the Ti foil (current collector), the drop cast of the polymer electrolyte layer (*ca*. 200 μm) and the LNMO_(100EP)_ cathode (*ca*. 20 μm). The Al cathode current collector was removed on top of the assembly to facilitate imaging of the cathode. The ohmic drop due to the electropolymer in the LNMO_(EP)_ increases the overvoltage and hence has an influence on the operating voltage of the microbattery.

**Figure 7:**
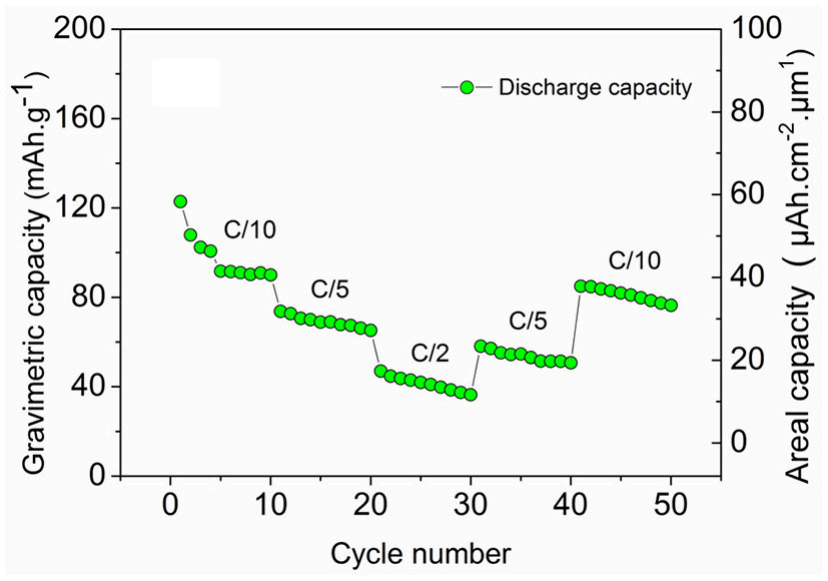
Discharge capacity of the microbattery TiO_2_ nts/Polymer/LNMO_(100EP)_ at multi-C rates.

Figure 7 shows the cycling performance of TiO_2_ nts/Polymer/LNMO_(100EP)_ at different C-rates for 50 cycles. At C/10, though there is a capacity decrease rapidly, it comparatively stabilizes at 89 mAh g^−1^ (41 μAh cm^−2^ μm^−1^). The microbattery delivers ~75 mA h g^−1^ (29 μAh cm^−2^ μm^−1^) at C/5 rate and ~47 mAh g^−1^ (15 μAh cm^−2^ μm^−1^) at C/2 rate. The microbattery retains comparable capacities of ~60 mAh g^−1^ (22 μAh cm^−2^ μm^−1^) at C/5 and ~85 mAh g^−1^ (37 μAh cm^−2^ μm^−1^) at C/10 rates. The energy and power densities obtained from such microbatteries are comparable to the performance of the prevalent 3D electrodes based microbatteries and commercial products (Pikul et al., 2013).

## Conclusions

Electrodeposition of PMMA-PEG electrolyte is studied into porous LNMO cathode electrode. SEM images reveal that the deposition follows and fills the porosity of the layer. These results have been supported by the impedance measurements suggesting the increase in the overall impedance with the formation of the insulating polymer thin layer with successive cycles of electropolymerization. Swagelok full-cell tests of the LNMO_(EP)_ vs. TiO_2_ nts revealed the capacity increase with the polymer thickness, a clear indication of a significant improvement of the electrode-electrolyte interface. Finally an all-solid-state microbattery has been fabricated using the polymer-coated LNMO_(100EP)_ as the cathode and TiO_2_ nts as the anode. Such a microbattery delivered an initial capacity of 122 mA h g^−1^ (57 μAh cm^−2^ μm^−1^), with a capacity retention of 72% up to 10 cycles. These kind of microbatteries are stable and can be tested at different kinetics over 50 cycles. The electrochemical performances of these microbatteries are comparable with other micropower sources involving different 3D nanostructured electrodes.

## Author Contributions

GS did experimental works, AG and AK discussed the results and participated to the writing, CL and TD supervised the work.

### Conflict of Interest Statement

The authors declare that the research was conducted in the absence of any commercial or financial relationships that could be construed as a potential conflict of interest.
